# Effect of spontaneous breathing trial on extubation in patients with acute exacerbation of chronic obstructive pulmonary disease under mechanical ventilation

**DOI:** 10.1186/s12873-022-00672-y

**Published:** 2022-06-21

**Authors:** Wenjing Liu, Hong Guo, Jing Wang, Fang Ding

**Affiliations:** 1 Department of Respiratory and Critical Care Medicine, Harrison International Peace Hospital Affiliated to Hebei Medical University, Hengshui, 053000 China; 2 Department of Neurosurgery, Harrison International Peace Hospital Affiliated to Hebei Medical University, 180 Middle Renmin Road, Hengshui, 053000 China; 3 Department of Gerontology, Harrison International Peace Hospital Affiliated to Hebei Medical University, Hengshui, 053000 China

**Keywords:** Spontaneous breathing trial, Acute exacerbation of chronic obstructive pulmonary disease, Invasive mechanical ventilation, Extubation

## Abstract

**Objective:**

To evaluate how spontaneous breathing trial (SBT) affects successful extubation and prognosis in acute exacerbation of chronic obstructive pulmonary disease (AECOPD) patients under mechanical ventilation.

**Methods:**

AECOPD patients under invasive mechanical ventilation were recruited into the study and divided into the SBT and non-SBT groups. SBT patients received SBT for 60 min before extubation, while non-SBT patients that met weaning criteria were immediately extubated without SBT.

**Results:**

A total of 64 patients were enrolled in analysis, including 32 in SBT group and 32 in non-SBT group. Patients in the two groups had similar baseline demographics and clinical characteristics (all parameters: *p* =  > 0.05). Four (12.5%) patients in the SBT group and 5 (15.6%) in the non-SBT group were reintubated in 48 h of extubation (*p* = 0.821). During the 28-day follow-up after extubation, 3 patients died, 1 (3.1%) in the SBT group and 2 (6.3%) in the non-SBT group (*p* = 0.554).

**Conclusion:**

Our findings indicate that SBT did not affect extubation success, in-hospital mortality, and 28-day survival in AECOPD patients under mechanical ventilation.

## Introduction

COPD (chronic obstructive pulmonary disease) is the 3^rd^ leading cause of death after ischemic heart disease and stroke. In China, COPD prevalence is as high as 15.5% [1]. Due to rising smoking rate in developing countries and aging populations in high-income countries, COPD prevalence is expected to rise in the next 40 years. It is projected that by 2060, > 5.4 million people may die of COPD and related diseases each year [2, 3].

AECOPD (acute exacerbation of COPD) is a major cause of hospitalization. In AECOPD patients, the grade of carbon dioxide retention can be caused by the aggravation of airflow. Severe AECOPD may cause pulmonary encephalopathy. Where noninvasive ventilation support cannot relieve CO_2_ retention, tracheal intubation with an invasive breathing machine may effectively support respiration in patients with AECOPD. Invasive intubation can ease patient breathing, ensure effective ventilation, improve airflow limitation, improve CO_2_ retention, and enhance the effectiveness of other treatments in patients with AECOPD [4, 5].

Spontaneous breathing trial (SBT) uses T-tube or minimal spontaneous breathing support in patients under invasive mechanical ventilation for short-term dynamic observation of patients’ ability to tolerate spontaneous breathing so as to assess the chance of successful weaning [6, 7]. Some studies suggested that 30–120 min SBT before weaning can effectively determine patient conditions before weaning. Others reported that reconnection to mechanical ventilation for 1 h after successful SBT may reduce the incidence of reintubation after weaning critically ill patients [8–12]. Some researchers suggested that SBT has no effect on extubation success in ICU (intensive care unit) patients [13, 14]. A systematic review on weaning indicated that there was no significant difference in duration of ventilation between patient used SBT and stepwise reduction in support [15]. However, there is no consensus on whether SBT is helpful during extubation in patients with AECOPD. Here, we investigated how SBT affects extubation success and prognosis in AECOPD patients under invasive mechanical ventilation.

## Patients and methods

### Patients

We retrospectively reviewed the medical records of AECOPD patients who underwent invasive mechanical ventilation at our hospital’s respiratory ICU from January 2020 to May 2020. Inclusion criteria were: > 18 years old, diagnosis of COPD with respiratory failure based on the GOLD (global initiative for chronic obstructive lung disease) criteria, endotracheal tube mechanical ventilation, and mechanical ventilation for > 48 h. Exclusion criteria were: central system diseases other than pulmonary encephalopathy, severe arrhythmia, cardiogenic shock, myocardial infarction, acute pulmonary embolism, pneumothorax, massive pleural effusion and lung malignant tumor, upper gastrointestinal perforation, obstruction, massive hemorrhage, recent digestive tract surgery, and tracheotomy.

This study was granted ethical approval by the ethics committee of Harrison International Peace Hospital. All participants gave written informed consent.

### Procedures

All patients were assessed according to the following readiness for weaning criteria [16]. SIMV (synchronized intermittent mandatory ventilation) and PSV (pressure support ventilation) were used in the initial stage of invasive mechanical ventilation. Ventilator parameters were then gradually adjusted to the patient’s condition. Invasive mechanical ventilation was done > 48 h before weaning. The timing of invasive mechanical ventilation removal was determined by: full wakefulness (Glasgow coma scale [GCS] score of 15, normal blood pH (7.35–7.45); presence of cough reflex; presence of pulmonary infection control (PIC) window which was characterized by improved sputum volume, sputum character, sputum color, core temperature of < 38.0 °C, normal white blood cell count, shrinkage of lung infiltration shadow, serum procalcitonin (PCT) levels of < 0.25 ug/L [17]; stabilization of arterial blood partial carbon dioxide (PCO_2_) pressure within a fluctuation of ± 20%; oxygenation index (arterial oxygen partial pressure divided by inhaled oxygen concentration) > 150–200; rapid shallow breathing index (RSBI) < 105 breaths/min/L; tidal volume > 5 mL/kg; minute ventilation > 0.1 L/kg; inspiratory pressure of ventilator 8–12 cm H_2_O; positive end-expiratory pressure (PEEP) ≤ 5–8 cm H_2_O; and respiratory rate 12–18 times/min.

SBT was given for 60 min using a T-piece to connect the external end of tracheal intubation. Continuous normal saline atomization was given to avoid airway drying, followed by extubation according to Practical Guidelines for Mechanical Ventilation [16]. In case of the following, SBT was immediately stopped and the patient placed on re-use of invasive mechanical ventilation: RSBI > 105 breaths/min/L, respiratory rate < 8 times/min or > 35 times/min, change of heart rate by > 20% or > 140 times/min and absence of new arrhythmia, tidal volume < 4 mL/kg, or oxygen saturation (SaO_2_) of < 0.90. RSBI is the ratio determined by the respiratory rate divided by the tidal volume in liters during an SBT. The respiratory rate of patients was monitored by the bedside ECG monitor. The tidal volume was monitored by the bedside portable pulmonary function instrument, with the blowing valve of the pulmonary function instrument connected with end of the T-piece.

Non-SBT patients who met weaning criteria were immediately extubated, and oxygen supplied by facemask. For all patients, blood gas analysis will be rechecked 1 h and 2 h after extubation, and not all patients were given noninvasive ventilatory (NIV) support within 2 h after extubation. The indications of NIV were: a) patient status: conscious, can remove airway secretions autonomously, shortness of breath (frequency > 25 times/min), auxiliary respiratory muscle participating in respiratory movement; b) blood gas index: pH ≤ 7.35 when breathing indoor air at sea level, arterial partial pressure of oxygen (PaO2) < 60 mmhg (1 mmhg = 0.133 kPa) with or without partial pressure of carbon dioxide (PaCO2) > 45 mmhg. The clinicians followed the same standardized operation process and extubation indications, and the extubation was operated by the same medical team, so it was considered that the bias introduced by process of weaning was marginal.

### Assessment

The following data at baseline were recorded: age, gender, BMI (body mass index), APACHE (acute physiology and chronic health evaluation) II score, comorbidity, pulmonary function classification, arterial blood gas analysis pH value, PCO_2_, heart rate, hemoglobin level, albumin levels, and body temperature. After extubation, the number of patients requiring reintubation (direct reintubation, reintubation after noninvasive ventilatory support) within 48 h, number of patients requiring noninvasive ventilatory support after extubation, and survival rate within 28 days of extubation were recorded.

### Statistical analysis

Statistical analyses were done on SPSS 24.0. Kolmogorov–Smirnov normal test was used to analyze data distribution. Normal distribution data were presented as mean ± SD. Variables data between groups were compared using t-test. Attributes data between groups were analyzed using Chi-square test. *P* < 0.05 indicated statistically significant differences.

## Results

### Baseline characteristics

**Fig. 1:**
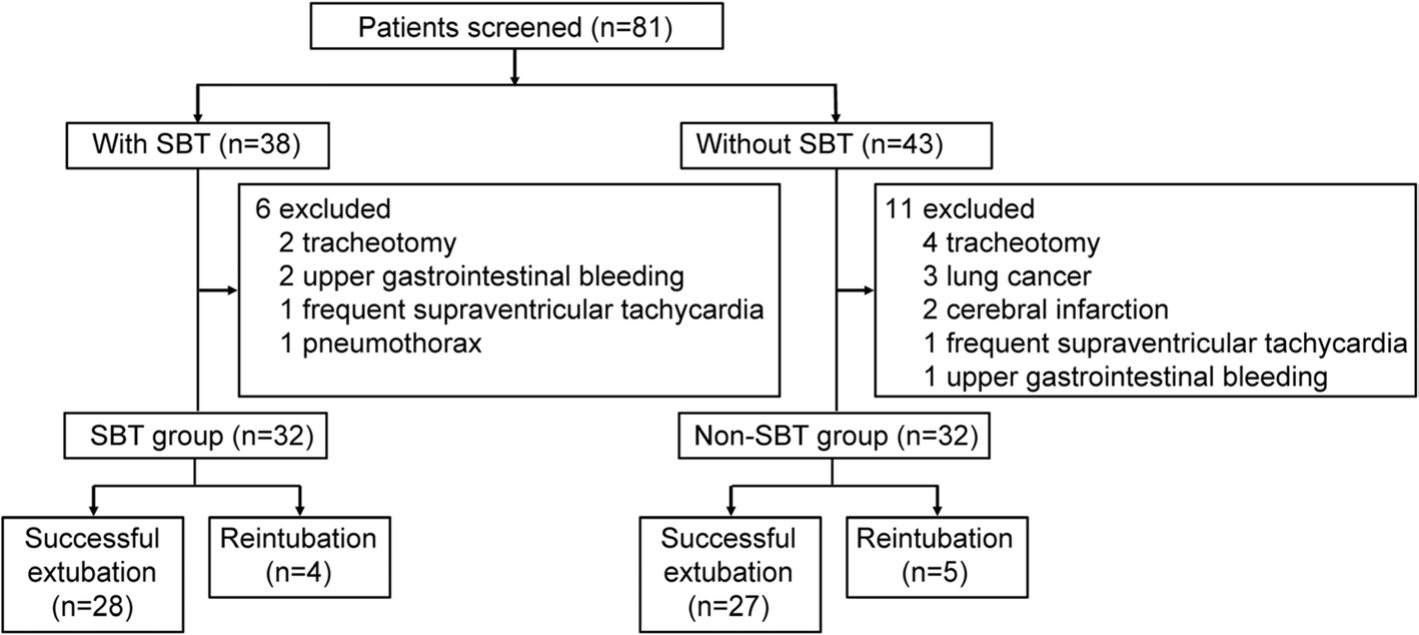
Patients disposition

A total of 64 patients were enrolled, including 32 patients in the SBT group and 32 patients in the non-SBT group (Fig. 1). Patients in the SBT group successfully underwent SBT. Baseline characteristics before invasive intubation of the 2 groups are shown on Table 1. The 2 groups did not differ significantly with regards to age, gender, BMI, and APACHE II score (all: *p* > 0.05). The number of prior invasive ventilation, duration of invasive ventilation, endotracheal tube size, and pulmonary function grades were similar in the 2 groups (all: *p* > 0.05). At baseline, the proportion of patients with listed comorbidities did not differ significantly between the 2 groups (*p* > 0.05). All patients had hypercapnia.

**Table 1:** Baseline characteristics before intubation

	**SBT group (*n* = 32)**	**Non-SBT group (*n* = 32)**	***P* value**
Age (years)	68.56 ± 7.63	70.03 ± 8.12	0.479
Male	24 (75.0%)	23 (71.9%)	0.465
BMI	24.05 ± 4.14	23.33 ± 3.56	0.458
APACHE II score	23.63 ± 7.64	25.87 ± 7.92	0.252
Prior invasive ventilation	14 (43.8%)	12 (37.5%)	0.611
Duration of invasive ventilation (days)	8.59 ± 2.83	9.31 ± 3.39	0.361
Size of endotracheal tube			
7.0 mm	8 (25.0%)	6 (18.8%)	0.545
7.5 mm	21 (65.6%)	24 (75.0%)	0.412
8.0 mm	3 (9.4%)	2 (6.3%)	0.641
Grading of pulmonary function (FEV1/FVC ratio)			
I (≥ 80%)	2 (6.1%)	2 (6.1%)	1.000
II (50–79%)	6 (18.6%)	5 (15.6%)	0.740
III (30–49%)	8 (25.0%)	9 (28.1%)	0.777
IV (< 30%)	16 (50.0%)	16 (50.0%)	1.000
Comorbid conditions			
Pneumonia	21 (65.6%)	16 (50.0%)	0.311
Cerebrovascular disease	3 (9.4%)	6 (18.8%)	0.474
Chronic kidney disease	6 (18.8%)	8 (25.0%)	0.736
Cardiovascular disease	7 (21.9%)	8 (25.0%)	0.768
Hypertension	5 (15.6%)	5 (15.6%)	1.000
Type II diabetes	3 (9.4%)	4 (12.5%)	0.689
Digestive system disease	2 (6.3%)	1 (3.1%)	0.554
Malignant tumor (extrapulmonary)	1 (3.1%)	0	0.313
Hypercapnia	32 (100%)	32 (100%)	/

Data are mean ± SD or n (%). *BMI* Body mass index, *APACHE II* Acute Physiology and Chronic Health Enquiry II, *FEV1* Forced expiratory volume in one second, *FVC* Forced vital capacity

### Patient clinical parameters measured before extubation

The pH value, PCO_2_, heart rate, body temperature, albumin level, hemoglobin level, systolic blood pressure, and oxygenation index before extubation, did not differ significantly (Table 2, *p* > 0.05).

**Table 2:** Patient parameters measured before extubation

	**SBT group (*n* = 32)**	**Non-SBT group (*n* = 32)**	***P* value**
Oxygenation index	307.95 ± 36.99	292.73 ± 29.78	0.492
pH	7.42 ± 0.04	7.42 ± 0.04	0.965
PCO_2_, mm Hg	43.97 ± 7.40	42.75 ± 5.66	0.503
Heart rate, beats/min	82.00 ± 7.41	81.63 ± 9.85	0.080
Body temperature, ºC	36.53 ± 0.56	36.69 ± 0.47	0.219
Systolic pressure, mm Hg	127.41 ± 11.99	126.16 ± 16.31	0.728
Albumin, g/L	30.15 ± 3.01	31.33 ± 2.95	0.118
Hemoglobin, g/L	120.38 ± 12.48	123.25 ± 11.58	0.343

Data are mean ± SD. *PCO*_*2*_ Pressure of carbon dioxide in arterial blood

### Successful extubation and reintubation

A total of 55 patients, 28 (87.5%) in the SBT group and 27 (84.4%) in the non-SBT group, were successfully extubated for ≥ 48 h (*p* = 0.719, Table 3). The other 9 patients (4, 12.5% in the SBT group and 5, 15.6% in the non-SBT group) were reintubated in 48 h of extubation (*p* = 0.821). Of these, 2 (1, 3.1% in the SBT group and 1, 3.1% in the non-SBT group) needed reintubation directly after extubation (*p* = 0.858), while 7 (3, 9.4% in the SBT group and 4, 12.5% in the non-SBT group) needed reintubation within 48 h of noninvasive ventilation (*p* = 0.867). A total of 22 patients (10, 31.3% in the SBT group and 12, 37.5% in the non-SBT group) received noninvasive ventilation in 48 after extubation (*p* = 0.599).

**Table 3:** Successful extubation and reintubation within 48 h in study groups

	**SBT group (*n* = 32)**	**Non-SBT group (*n* = 32)**	***P* value**
Successful extubation	28 (87.5%)	27 (84.4%)	0.719
Reintubation	4 (12.5%)	5 (15.6%)	0.821
Direct Reintubation	1 (3.1%)	1 (3.1%)	0.858
Reintubation after noninvasive ventilation	3 (9.4%)	4 (12.5%)	0.867
Noninvasive ventilation after extubation	10 (31.3%)	12 (37.5%)	0.599
In-hospital death	0	0	1.000
Death within 28 days	1 (3.1%)	2 (6.3%)	0.554

Data are n (%)

There were no deaths during hospitalization. During the 28-day follow-up, 3 patients (1, 3.1% in the SBT group and 2, 6.3% in the non-SBT group) died after discharge (*p* = 0.554).

## Discussion

This study examined how pre-extubation SBT affects extubation success and prognosis in AECOPD patients under invasive mechanical ventilation. In the patients who received pre- extubation SBT, 28 (87.5%) were successfully weaned and did not need reintubation within 48 h, while 27 (84.4%) patients in the non-SBT group were successfully weaned (*p* = 0.719). Death rate within the 28-day follow-up was similar between the 2 groups (3.1% vs 6.3%, *p* = 0.554). These findings indicate that pre-extubation SBT is not necessary in AECOPD patients under invasive mechanical ventilation.

Pulmonary function analysis revealed that most patients in the SBT (75%) and non-SBT (78.1%) group had pulmonary function grades III-IV, suggesting that the worse the pulmonary function, the likelier it is to receive invasive mechanical ventilation due to acute disease exacerbation. However, correlation between pulmonary function grade and SBT needs to be studied in larger cohorts.

Here, T-tube use in the SBT group for 60 min was chosen based on a recent meta-analysis that found that T-tube and pressure support ventilation did not differ significantly with regards to the success of weaning, duration of hospital stay, and in-hospital mortality [18]. For patients with poor pulmonary function and difficult breathing after extubation, noninvasive ventilation support, including noninvasive ventilation and high-flow nasal oxygen (HFNO) should be timely [19]. It was considered that patients in SBT group will need more breath during SBT before extubation, resulting in respiratory muscle fatigue. Thus, noninvasive ventilation support can relieve respiratory muscle fatigue and aid successful extubation.

There is no consensus on the effect of pre-extubation SBT on patients’ prognosis. Wang et al. reported that pre-extubation SBT has no significant effect on the prognosis of critically ill patients [13]. Fernandez et al. suggested that extubation success can be improved by giving the same level of pressure support for 1 h between SBT completion and extubation [12]. Noninvasive monitoring of some related indicators may also be used to predict risks introduced by extubation. For example, Dubo et al. reported that early CVP (central venous pressure) after SBT initiation may indicate elevated risk of extubation failure [20]. Keyal et al. suggested that patients with invasive mechanical ventilation who successfully completed SBT should be guided by arterial blood gas monitoring to improve extubation success [21]. Ghosh et al. found that a cumulative fluid balance of ≥ 3490 mL during mechanical ventilation may increase the risk of extubation failure [22]. Here, we find that the risk of extubation failure and mortality within 28 days were similar in the SBT and non-SBT group.

This study is limited by its single-center design, limited cohort size, relatively short prognosis follow-up, and its retrospective nature, which limited control over exposure factors, covariates, and potential confounders. Thus, further prospective multicenter randomized controlled trial is needed to confirm our study results and give the highest level of evidence in clinical practice.

In conclusion, our findings show that SBT does not affect extubation success, in-hospital mortality, and 28-day survival after extubation of AECOPD patients under mechanical ventilation. Doctors should actively standardize extubation procedures and strengthen cooperation between patients and caregivers to improve extubation success, reduce mortality and improve patients’ quality of life.

## Data Availability

All data generated or analysed during this study are included in this published article.
